# Configuring thermal ablation volumes for treatment of distinct tumor shapes: a repeatability study using a robotic approach

**DOI:** 10.3389/fonc.2024.1463686

**Published:** 2024-12-20

**Authors:** Milica Bulatović, Jan Hermann, Pascale Tinguely, Iwan Paolucci, Stefan Weber

**Affiliations:** ^1^ ARTORG Center for Biomedical Engineering Research, University of Bern, Bern, Switzerland; ^2^ Clinical Service of HPB Surgery and Liver Transplantation, Royal Free Hospital, NHS Foundation Trust, London, United Kingdom; ^3^ Department of Interventional Radiology, The University of Texas MD Anderson Cancer Center, Houston, TX, United States

**Keywords:** liver cancer, tumor shape, perivascular, peribiliary, tissue-sparing, microwave ablation, robotic navigation, treatment personalization

## Abstract

**Objectives:**

In the current clinical practice of thermal ablation treatment for liver tumors, achieving consistent and effective clinical outcomes across tumors of varying shapes, sizes and locations remains challenging. The aim of this study was to evaluate the repeatability of a novel robotic approach for configurable ablation of distinct tumor shapes and compare it to the standard ablation technique for creating ellipsoidal ablation volumes.

**Materials and methods:**

The repeatability was evaluated in terms of width variability in created ablation volumes. Using a robotic navigation platform, custom ablation profiles configured with power, time, and distance parameters were designed to create four distinct ablation shapes. The profiles were applied for microwave ablation in a tissue-mimicking liver model. For comparison of ablation shape variability, six standard ellipsoidal shapes were created using the standard ablation technique by configuring power and time parameters. For each sample, the resulting ablation area was segmented, and the resulting shape width and length were calculated at the measurement points. Width variability was calculated as the median of the absolute pairwise differences in width at each measurement point, and *configurable* versus *standard* ablation shapes were compared using the Mann–Whitney U test.

**Results:**

All tissue-mimicking samples were successfully ablated using both configurable (n = 48) and standard ablation technique (n = 35). Study findings revealed noninferiority regarding repeatability of created ablation shapes using the robotic platform for configurable ablation, compared to created standard ellipsoidal ablation shapes (p < 0.001, 95% CI ≤ -0.05 mm, Δ = -0.22 mm). Median repeatability of created configurable shapes was 1.00 mm, and for standard shapes 1.22 mm. Maximal repeatability for both groups was below 3 mm.

**Conclusion:**

The repeatability of configurable ablation shapes was observed to be noninferior to the standard ablation shapes. Achieving configurable ablation volumes underscores the potential to advance personalization of thermal ablation treatment and broaden its applicability to distinct tumor cases. *In-vivo* validation is needed for evaluation of the clinical implications of this novel treatment technique.

## Introduction

**Figure 1:**
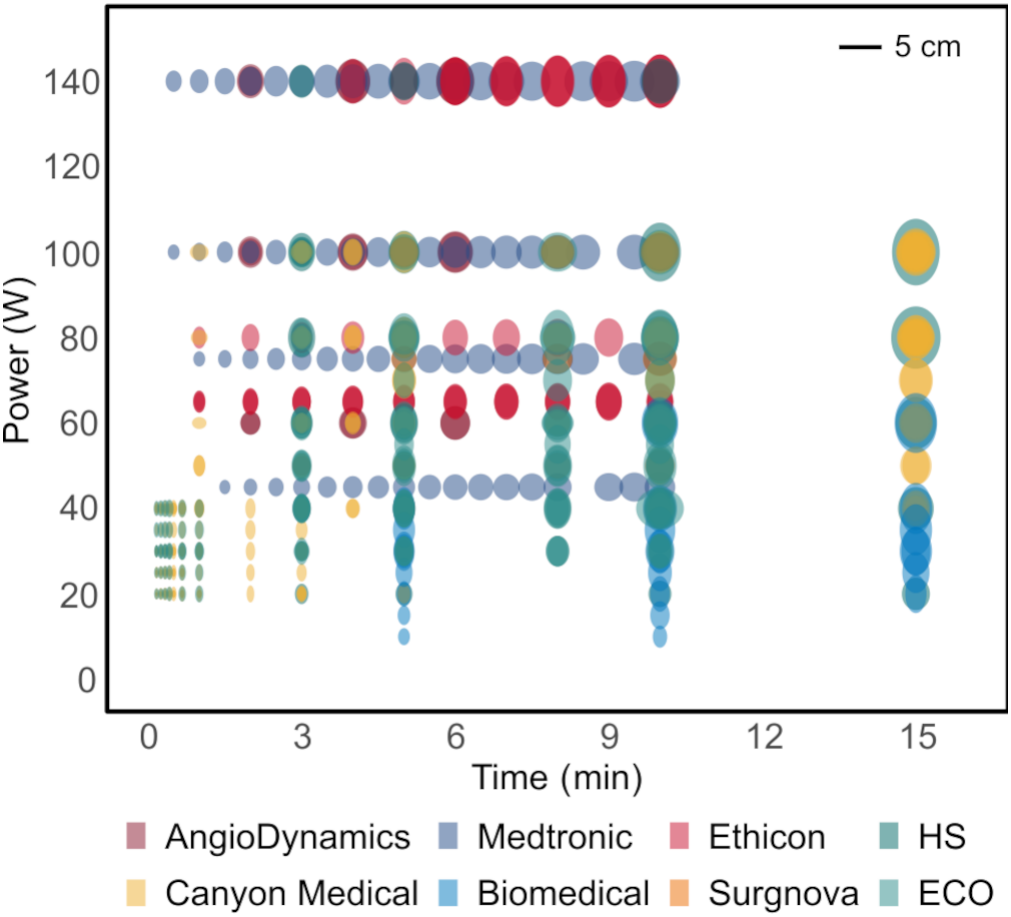
Microwave ablation catalogues depicting available ablation shapes using state-of-the-art ablation devices in *ex-vivo* animal tissue models. All the shapes are ellipsoidal, optimal for treatment of small spherical tumors.

The outcome of thermal ablation treatment of malignant liver tumors is highly variable (1). Small and spherical tumors (≤ 3–5 cm in diameter) can benefit the most from this treatment, since larger tumors are often associated with reduced technical efficacy and higher local tumor progression rates (2–5). Moreover, for irregularly shaped tumors and tumors in proximity to major hepatic vessels and central bile ducts, the therapeutic effectiveness and safety are often compromised (6, 7). Unwanted effects of large ablation areas can manifest in an excessive treatment area with potential thermal injury to neighboring organs or major intrahepatic structures, or an unnecessary loss of surrounding healthy liver parenchyma (8). The present treatment limitations arise mainly from technical constraints, since predominantly ellipsoidal ablation volumes up to 5 cm in diameter are available with commercial ablation devices (**Figure 1**). This set of ablation shapes does not allow precise tumor ablation in critical locations in the liver nor in the case of irregularly shaped tumors, which may restrict the applicability of thermal ablation treatment in these situations.

Traditionally, the choice of treatment strategy for malignant liver tumors depends on factors such as tumor size, number of nodules and proximity to vascular and biliary structures, rather than tumor shape (9, 10). However, the value of preserving healthy liver parenchyma has been shown through certain liver-directed therapies. For instance, non-anatomical liver resection, stereotactic body radiation therapy and irreversible electroporation can all treat tumors closely to their shape with sufficient margins (11–13). In single-probe thermal ablation, two techniques have been routinely used in the clinical practice to create more complex ablation shapes. The overlapping ablation technique (OAT) aims to provide complete coverage of larger liver tumors by sequential or simultaneous ablation of overlapping ablation volumes. The sequential approach results in a smaller, but more controllable ablation volume, whereas the simultaneous approach can achieve a large, but unpredictable ablation volume (14, 15). Furthermore, in other organs such as the thyroid, the moving-shot technique (MST) has been accepted over the past decade for treatment of thyroid nodules (16). This technique was defined as unit-by-unit ablation of thyroid nodules achieved by manual retraction of the ablation probe. In contrast to the fixed-electrode technique (FET), most commonly used for ablation of liver tumors, the MST emerged as a safer alternative aiming to prevent thermal injury to neighboring critical structures in the thyroid gland (17, 18).

The aim of this work was to remove the limitations imposed by the current state of ablation technology. The main objective was to extend the potential of thermal ablation to non-spherical and critically located tumors, by providing a comprehensive, personalized, and configurable treatment model. The model was described in a proof-of-concept study conducted by the authors in a tissue-mimicking liver model (19). It is based on a technique of configuring ablation volume shapes by robotically retracting the ablation probe along its axis while modulating the power over time and distance. The present experimental study aimed to compare the repeatability of ablation volumes created with the configurable ablation technique against the standard ablation technique, which produces mainly ellipsoidal ablation volumes. The hypothesis was that the repeatability of configurable ablation shapes is noninferior to the repeatability of the standard shapes in the tissue-mimicking model.

## Materials and methods

### Concept

The idea and the principles of a configurable thermal ablation treatment model was previously described by Paolucci et al. (19) The proposed treatment model aims to bring patient-specific solutions by means of dynamic thermal energy delivery. In this technique, the ablation probe is robotically retracted along its axis while the ablation power is modulated over time and distance, such that each segment of the lesion receives the required energy dose according to the previously calculated treatment plan. The novelty of this approach is in creating personalized ablation shapes tailored to the individual tumor morphologies. The theoretical application criteria for the proposed treatment model in the liver are small and medium sized tumors (≤ 5 cm), including irregularly shaped tumors located in complex locations, such as in proximity to the liver capsule or larger intrahepatic vascular structures.

### Experimental setup

**Figure 2:**
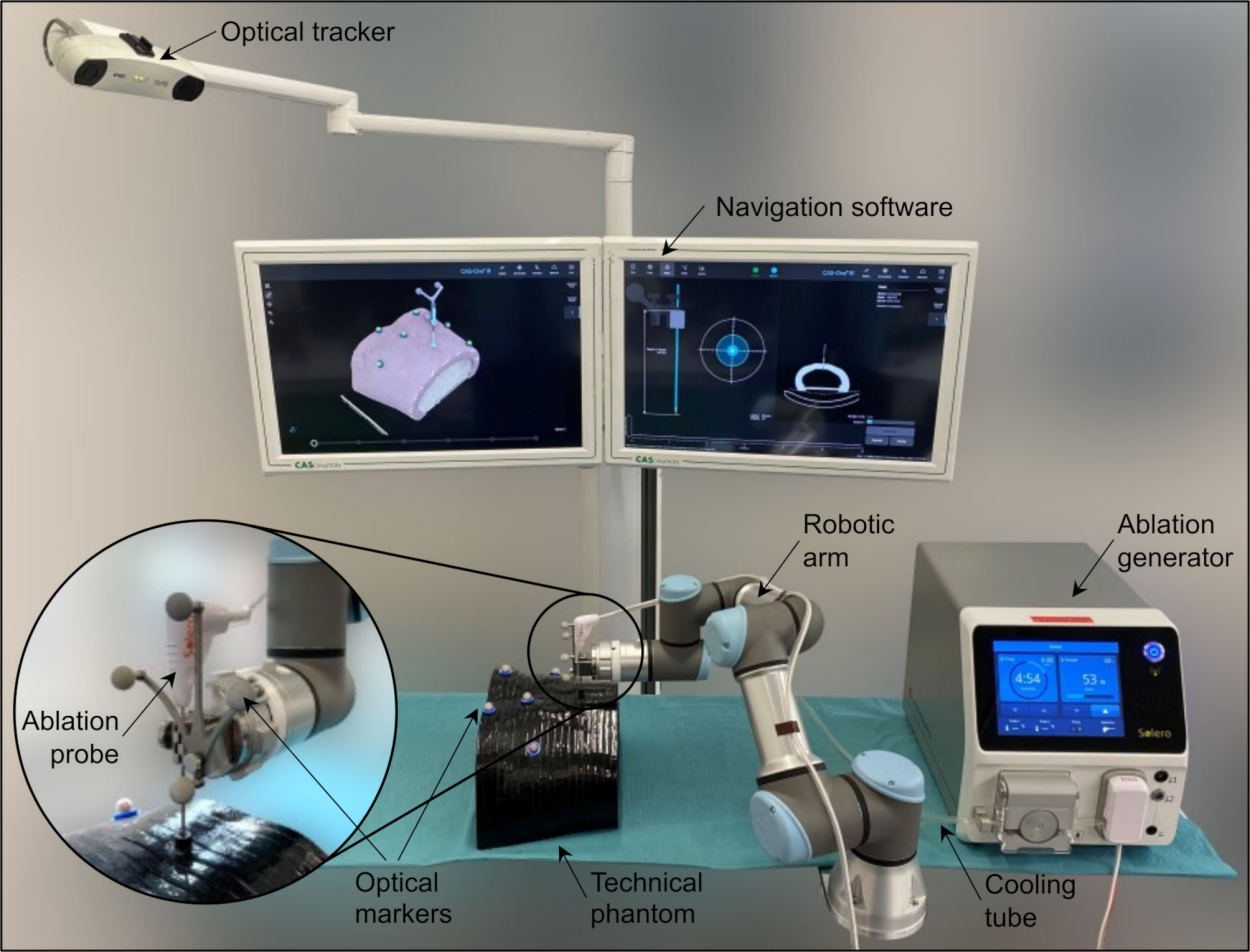
Experimental setup including a navigation system with optical tracking, a robotic arm, a microwave ablation device with liquid cooling system and an ablation probe, a technical phantom mimicking the human abdomen shape with optical markers, and a tissue-mimicking specimen placed inside the phantom.

Respective to the presently used clinical solutions for image-based navigation and treatment planning, the core components of the system are (**Figure 2**): a stereotactic image-guidance system with optical tracking (CAS-One IR, CASCINATION, Bern, Switzerland), a 6-DoF robotic arm with an optically tracked end-effector (UR3e, Universal Robots, Odense, Denmark), and an ablation system with a microwave generator and a water-cooled ablation probe (Solero, AngioDynamics, Latham, NY, USA). By coupling these components, the system is able to deliver a customized thermal ablation treatment plan. In the experimental setup, the “patient” was represented by a carbon-fiber abdominal phantom with six optical tracking markers and a dedicated inner space to place the tissue-mimicking samples.

### Software implementation

The robot control software was implemented in the ROS2 framework (ROS2 Humble, Robot Operating System, Stanford Artificial Intelligence Laboratory, Stanford, CA, USA) on a real-time Linux computer (Ubuntu 22.04 5.15.96-rt61). In addition to the custom developed software solutions (e.g. ablation profile trajectory planning), freely available libraries were used: Universal Robots’ ROS2 driver for robot control, and MoveIt2 library for trajectory execution. The communication with the navigation system was established via ethernet connection using ZMQ messaging protocol (version 4.3.1). On the navigation system, a software workflow was implemented and integrated into the CAS-One IR software (CASCINATION, Bern, Switzerland). The user could select the desired ablation shape in the planning step (**Table 1**) and send commands to control the robot.

**Table 1:** Selected ablation profiles.

Ablation profile	Power (W)	Time (s)	Distance (mm)	Energy (kJ)	Shape
*Standard*					
OVAL-60W-120s	60	120	0	7.2	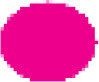
OVAL-60W-240s	60	240	0	14.4	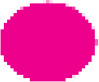
OVAL-60W-360s	60	360	0	21.6	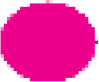
OVAL-100W-120s	100	120	0	12.0	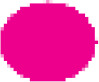
OVAL-100W-240s	100	240	0	24.0	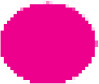
OVAL-100W-360s	100	360	0	36.0	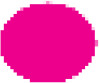
*Configurable*					
LONG-60W-600s	60	600	50	36.0	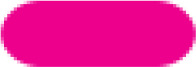
HOUR-60W-600s	60	260, 80, 260	0, 40, 0	36.0	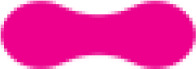
TEAR-60W-600s	60	360, 210, 30	0, 30, 15	36.0	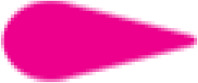
PEAR-60W-600s	60	100, 200, 300	25, 12.5, 12.5	36.0	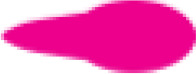

### Ablation profile design and selection

Ablation profiles were designed as series of intervals in which the ablation probe was moving with certain settings with respect to power, time, and distance. Previous feasibility experiments revealed that an ablation shape can be configured with slow ablation probe retraction speeds (< 1 mm/s), provisional probe trajectory lengths and a stable power output (19). To better understand and facilitate the ablation profile design process, a simple numerical model was used to simulate heat propagation and visualize prospective ablation shapes. The model was based on the heat conduction equation in an isotropic material and could be tuned for individual ablation devices and tissue models (20).

In this study, four distinct configurable ablation profiles were designed according to four distinct tumor shapes: (A) elongated/LONG, (B) hourglass/HOUR, (C) teardrop/TEAR and (D) pear-shaped/PEAR (**Table 1**, *Configurable*). These shapes aimed to accommodate different clinical scenarios and represent different possibilities of ablation shape configuration, which could be combined to obtain the targeted ablation shape. For all four profiles, the total duration was 10 minutes, and the ablation power setting was fixed at 60W. In previous experiments, 60W provided the most predictable shape outcomes using the same ablation device (19). A total of 35 samples were ablated in the tissue-mimicking model. For the standard ablation shapes, six ablation profiles were selected from the manufacturer’s catalogue to ablate 48 tissue-mimicking samples (**Table 1**, *Standard)*.

### Sample preparation

The tissue-mimicking model used in this study has an irreversible thermochromic property which makes it permanently change its color from off-white to magenta when heated above the coagulation threshold temperature (60°C) (21, 22). This model, however, does not have a coagulative property, hence three-dimensional evaluation of the resulting ablation shapes using CT images was not possible. The tissue samples were prepared by mixing distilled water, acrylamide/bis-acrylamide 40% (19:1), Kromagen Magenta MB60-NH ink, ammonium persulphate, and tetramethylethylenediamine under magnetic stirring (76.5% v/v, 17.5% v/v, 5.6% v/v, 0.2% v/v, 0.2% v/v). The solution was then poured into cylindrical containers with caps (D54 mm × H90 mm) to stabilize. Prior to ablation, the samples were kept in a water bath at 37°C for at least two hours to temper. After ablation, the samples in their closed containers were placed back into the tempered water bath until the post processing step.

### Experimental workflow

For each sample, the experiment was conducted as follows. First, the ablation probe trajectory planning and the ablation shape selection were done on the robotic platform. Secondly, the robot end-effector was robotically aligned to the entry position of the planned trajectory, and the ablation probe was inserted either robotically or manually at the target position, depending on the trajectory length and the workspace constrictions. Thirdly, the ablation was performed with robotic position-controlled probe retraction and manual ablation power modulation. Lastly, the resulting ablation volume was measured in a semi-automatic computational measurement workflow (see *Sample post-processing and analysis*).

During the ablation process, the output power of the ablation device was captured every 30 s, and the ablation probe position was recorded every 0.05 s by the tracking camera. Due to the quantization error, the robot could not move with linear speeds less than 1 mm/s. For this reason, if the commanded ablation probe speed at a certain ablation profile interval went bellow 1 mm/s, the interval was discretized in 2 mm distance steps with 2 s retraction periods plus a waiting period, such that the mean speed per segment resulted in the desired speed. The ablation probes were gathered from previous clinical interventions, disinfected with ethanol, and reused in the study. Four ablation probes were used in total. The length of the probes was 19 cm, the diameter was 15 gauge and the length from the probe tip to the feed zone was 18 mm. Due to heating from the ablation probe, the cooling liquid was replaced after each ablation cycle (6 min) and stored in 1 l bottles at temperatures 0–5°C prior to use.

### Sample post-processing and analysis

**Figure 3:**
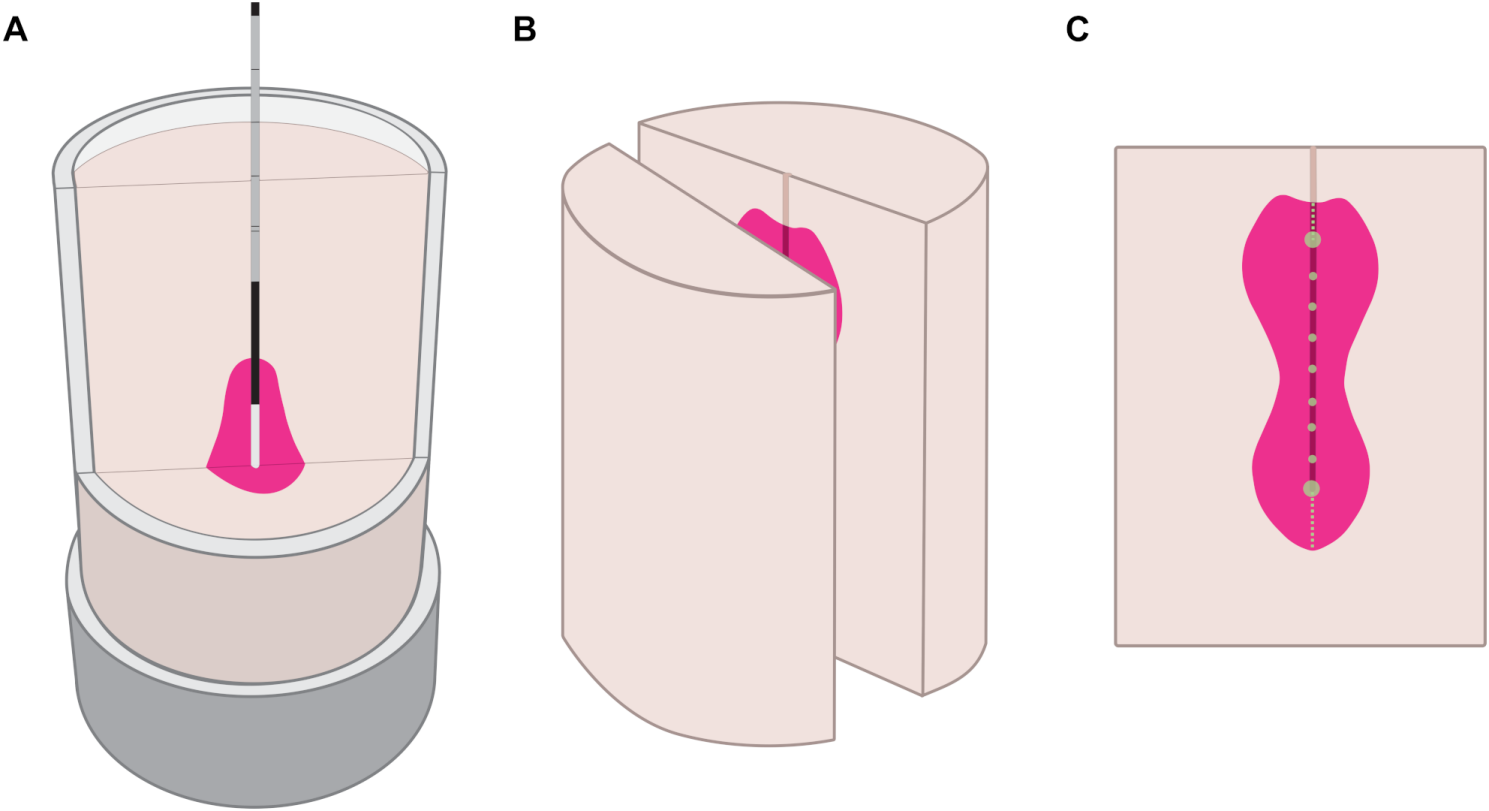
Experimental workflow represented in three phases. Ablation process in the tissue-mimicking sample **(A)**, post-ablation sample cut along the probe axis **(B)**, ablation sample cross-section with width measurements at green points, length measurements along dashed lines **(C)**.

After the ablation procedure, each sample was cut in half along the probe axis (**Figures 3A, B**). Then, an optical image of the cross-section was taken, and the measurement scale was determined using ArUco markers placed in the three corners around the sample holder. The ablation shape was segmented using a color-based approach (OpenCV 4.9, Python 3.12). Similar to the Otsu’s segmentation method, the segmentation algorithm classified each pixel as foreground (ablated, magenta color) or background (non-ablated, off-white color). The classification was based on a weighted Euclidean distance of the pixel’s HSV coordinates (hue, saturation, value) to the median foreground and background HSV coordinates in each image. The classification threshold was set as the color equally distanced between the two median colors, such that the segmentation boundary always lied in the middle of the color gradient between the ablated and non-ablated areas. Following the segmentation, the variation of the ablation shape contour was quantified: the widths and the lengths were measured along the short and the long axes from the ablation profile starting point to the end of the trajectory in 5 mm measurement steps (**Figure 3C**). For standard ablation shapes, there was only one measurement point for all six ablation profiles, and for configurable ablation shapes, the number of measurement points depended on the length of the ablation probe trajectory.

### Evaluation metric and statistical analysis

The median and the interquartile range (IQR) of ablation widths and lengths were calculated to estimate the distribution at each measurement point. The metric to compare the repeatability in terms of width variability of created ablation shapes was defined as the median of the absolute pairwise differences in width at each measurement point. The median of this metric is the the Rousseeuw-Croux *Sn* estimator, a robust non-parametric method for quantifying variability (23). Like *Sn*, the chosen metric is resistant to outliers and is suitable for analyzing asymmetric distributions. The Mann–Whitney U test was applied to test noninferiority regarding repeatability of produced configurable versus standard ablation shapes. The threshold for statistical significance was set to α < 0.05. All statistical calculations were done using R statistical software (R, R Foundation for Statistical Computing, Vienna, Austria).

### Sample size

To determine the required sample size of configurable versus standard shapes, a noninferiority power analysis was conducted using pilot data collected in this study and the results of the previous feasibility study (21). In the feasibility study, single width measurements were taken from 27 configurable shapes of three ablation profiles. The pooled standard deviation of the measured ablation shape widths was 1.1 mm. The pilot data in this study included 8 standard shapes of two ablation profiles, for which the pooled standard deviation measured 0.5 mm. By assuming normal distribution of ablation widths for both standard and configurable shapes, large number of datapoints could be generated by using the calculated standard deviations and an arbitrary mean. The ratio of points generated for the two groups was 0.5 (standard: configurable), since the configurable shapes have more measuring points than the standard shapes (see *Sample post-processing and analysis*). The sample size was calculated by generating corresponding number of samples in each iteration, until the Mann-Whitney U test returned 100% probability of reaching statistical significance (p = 0.05). The noninferiority margin was set to 1.0 mm. The number of repeatability measurement samples was calculated to be 25 for standard and 125 for configurable shapes. For redundancy, to account for potential outliers due to technical or experimental limitations, the study was conducted on a total of 48 standard and 35 configurable shape tissue-mimicking specimens, resulting in 42 and 350 repeatability measurement points respectively.

## Results

**Figure 4:**
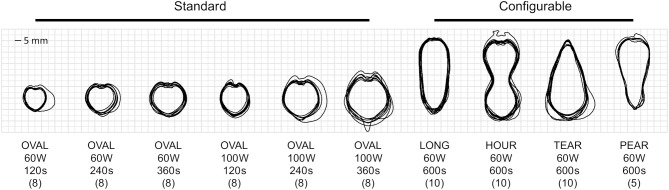
Overlay of segmented contours for produced standard (n = 48) and configurable (n = 35) ablation shapes, along with shape descriptions, planned power, duration, and sample sizes (in brackets).

All 48 samples of standard ablation shapes, and all 35 samples of configurable ablation shapes were successfully prepared, ablated and post-processed. Three additional samples had to be discarded since the ablation probe had to be replaced, mainly due to issues related to the cooling system (tubing tear or clog). Segmented ablation contours from the sample cross-sections were used for the statistical analysis (**Figure 4**). Visually, all the ablation shapes showed similar amount of repeatability around their contours. The standard ablation shapes (**Figure 4**, OVAL) appeared to be rather heart-shaped than ellipsoidal, as suggested in the manufacturer’s catalogue. The configurable ablation shapes appeared as simulated (**Figure 4**, LONG–PEAR). An explanatory video showcasing the process of configurable ablation for one of the shapes is provided in the **Supplementary Material**.

**Figure 5:**
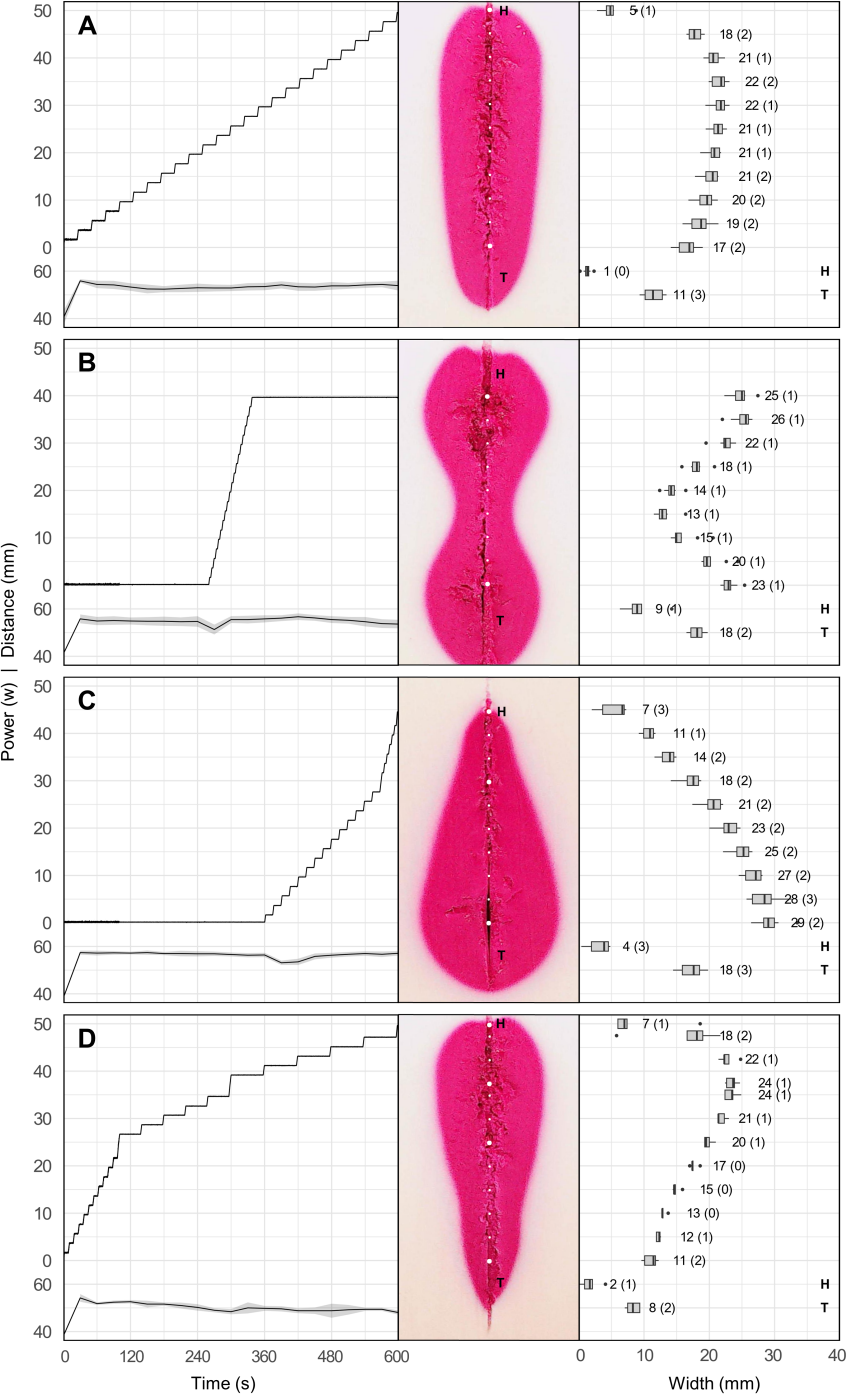
Configurable ablation shapes including: elongated/LONG **(A)**, hourglass/HOUR **(B)**, teardrop/TEAR **(C)**, and pear-shaped/PEAR **(D)**. Each panel shows the applied ablation profile containing distance travelled by the ablation probe (left, top curve) and output power from the ablation generator (left, bottom curve); resulting ablation shape in the tissue-mimicking model marked with measurement points (middle), ablation widths (median, IQR in brackets) at specified measurement points, and ablation lengths at head (H) and tail (T) areas (right).

For the configurable shapes, the applied ablation profiles (**Figure 5**, left), the resulting ablation shapes (**Figure 5**, middle), and the measured ablation widths (**Figure 5**, right) are shown. The recorded distances travelled by the ablation probe were step functions due to profile discretization. However, the average speed per individual distance interval (**Figure 5**, marked between the dashed lines) corresponded to the planned probe speeds (**Table 1**, distance over time). The recorded output power values varied within 85–90% of the planned power values (**Table 1**, power). Sample illustrations of the resulting ablation shapes are shown in the tissue-mimicking liver model, where the ablated area was stained in magenta color, and non-ablated area in off-white color. Ablation probe marks were visible along the centerline of the samples, where the white dots correspond to the measurement points (spaced in 5 mm increments). The letters, *H* for head and *T* for tail, indicate the areas outside of the planned probe trajectories. It should be noted that the tail areas were always greater than zero, even in cases where the probe was constantly in motion (**Figures 5A, D**). On the contrary, the head areas were negligible in those cases, and only prominent when the ablation probe was static for certain amount of time (**Figures 5B, C**). Regarding median ablation shape width, the elongated shape measured 22 mm at the widest point, the hourglass shape 26 mm at the widest point and 13 mm at the narrowest point, the teardrop shape 29 mm at the widest point and 7 mm at the tip, and the pear ablation shape 24 mm at widest point and 11 mm at the tip. The width spread (interquartile range, IQR) at each measurement point maintained roughly an even trend in all ablation shapes, it ranged 1–2 mm for the elongated shape, 1 mm for the hourglass shape, 1–3 mm for the teardrop shape, and 0–2 mm for the pear ablation shape (**Figures 5A–D**). Similarly, this also applied to the length spread (IQR) at the head and tail, where the measurements ranged 0–3 mm, 1–2 mm, 3 mm, 1–2 mm respectively.

**Figure 6:**
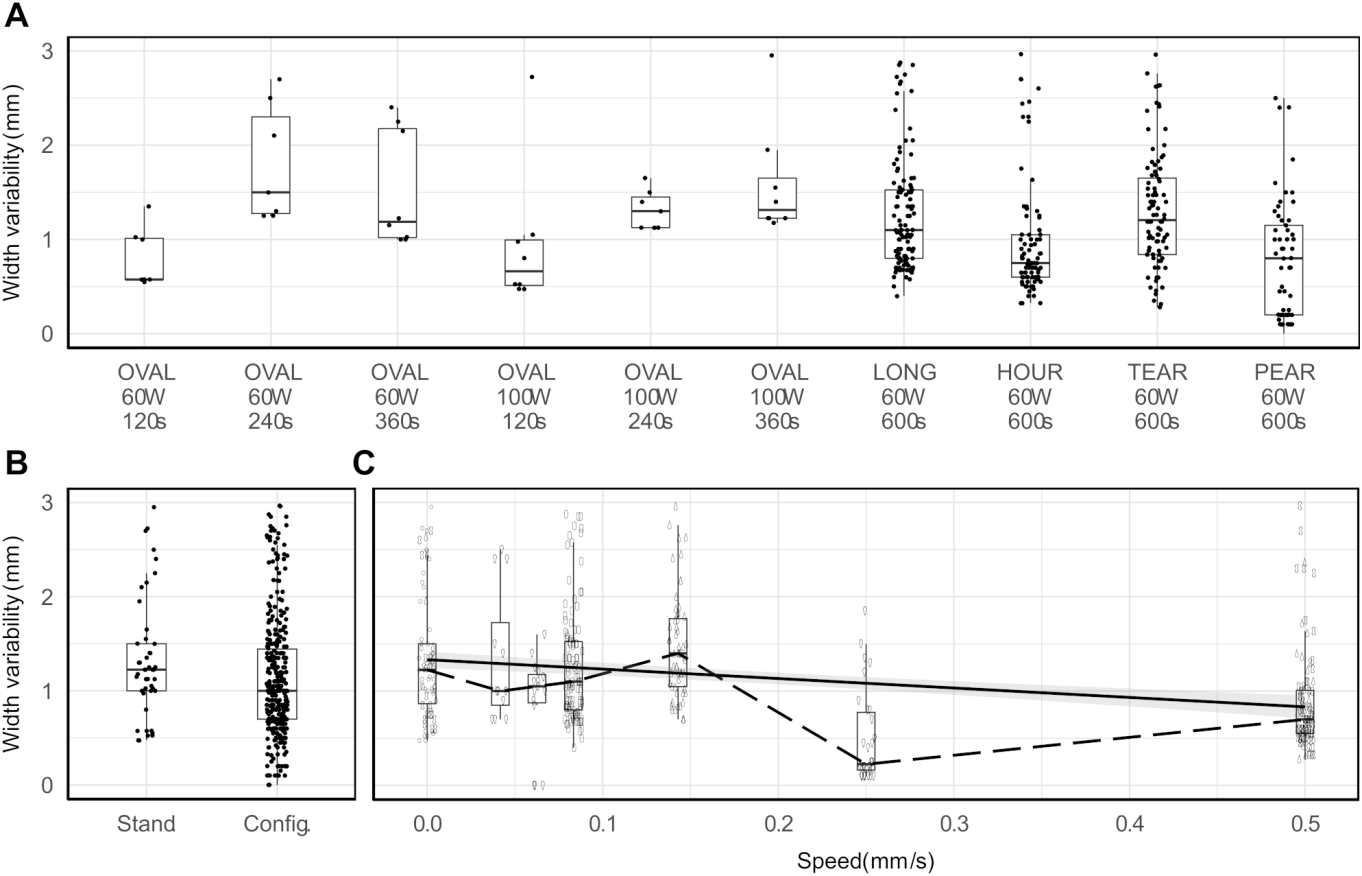
Repeatability measured in terms of ablation width variability per ablation shape **(A)**, per ablation shape type **(B)**, and per changes in ablation probe speed (solid line: fitted linear regression, dashed line: median values) **(C)**.

The repeatability of configurable ablation shapes was noninferior to standard ablation shapes for all ablation shape pairs (one standard, one configurable) (**Figure 6A**). The median (maximal) repeatability measured as width variability in 95% of observed shapes ranged 0.57–1.50 mm (1.35–2.95 mm) in standard and 0.75–1.20 mm (2.50–2.96 mm) for configurable shapes. Furthermore, the repeatability was observed to be noninferior in configurable (median 1.00 mm, IQR 0.70–1.44 mm) than in standard shapes (median 1.22 mm, IQR 1.00–1.50 mm), as determined by the Mann-Whitney test (p < 0.001, 95% CI ≤ -0.05 mm, Δ = -0.22 mm) (**Figure 6B**). Lastly, a linear regression analysis indicated that higher probe speeds were associated with a decrease in repeatability of the observed shapes (p < 0.001, β = -1.00 mm/s, 95% CI = [-1.33, -0.67] mm, R^2^ = 0.08). However, a low R^2^ value showed that other factors aside from probe speed may also have a significant impact on the repeatability (**Figure 6C**).

**Figure 7:**
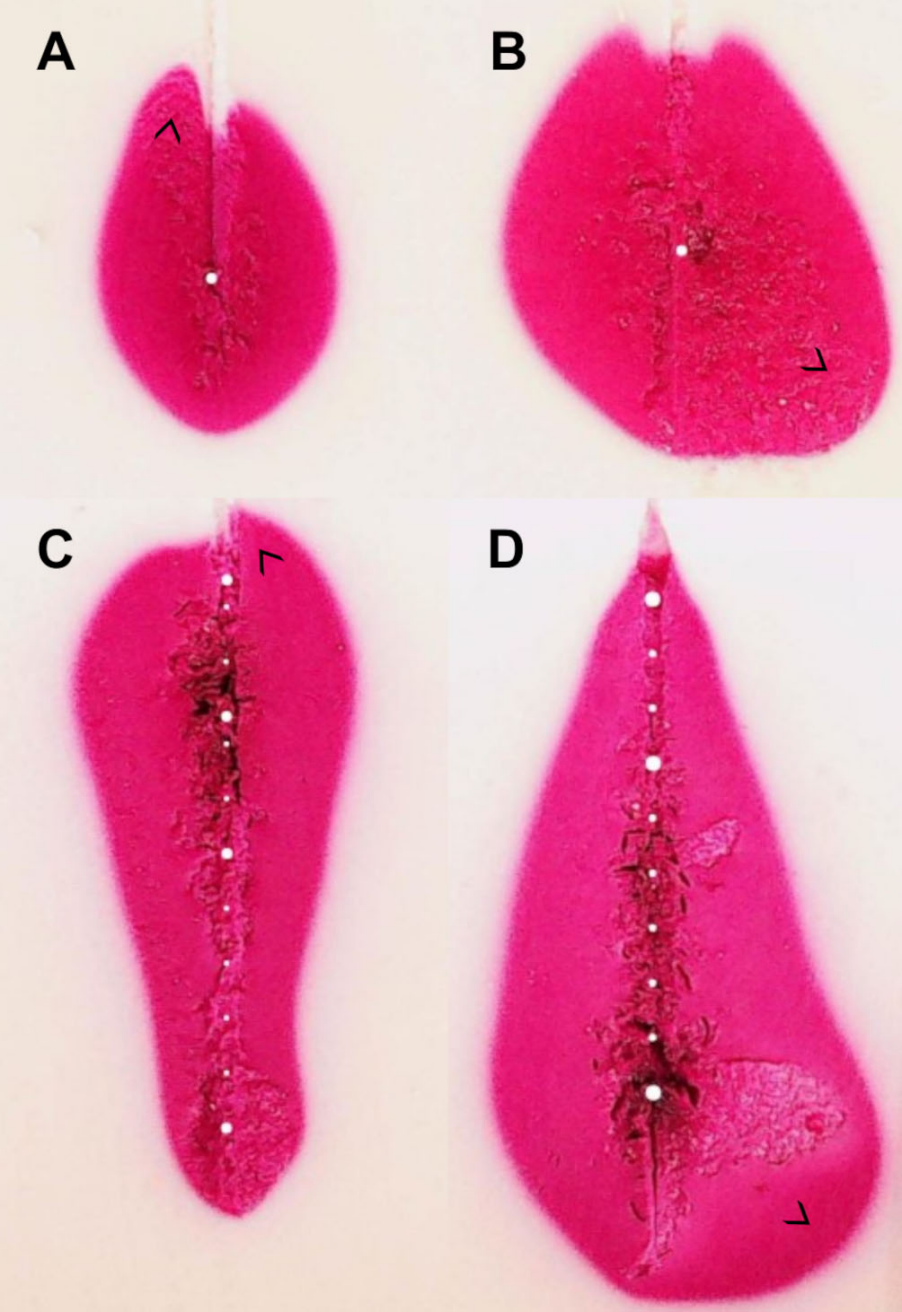
Examples of ablation shape outliers **(A–D)**. Discrepancies can appear in different locations along the ablation contour as marked with arrows. Similar effects are observed in real human tissue and ex-vivo animal tissue.

**Figure 8:**
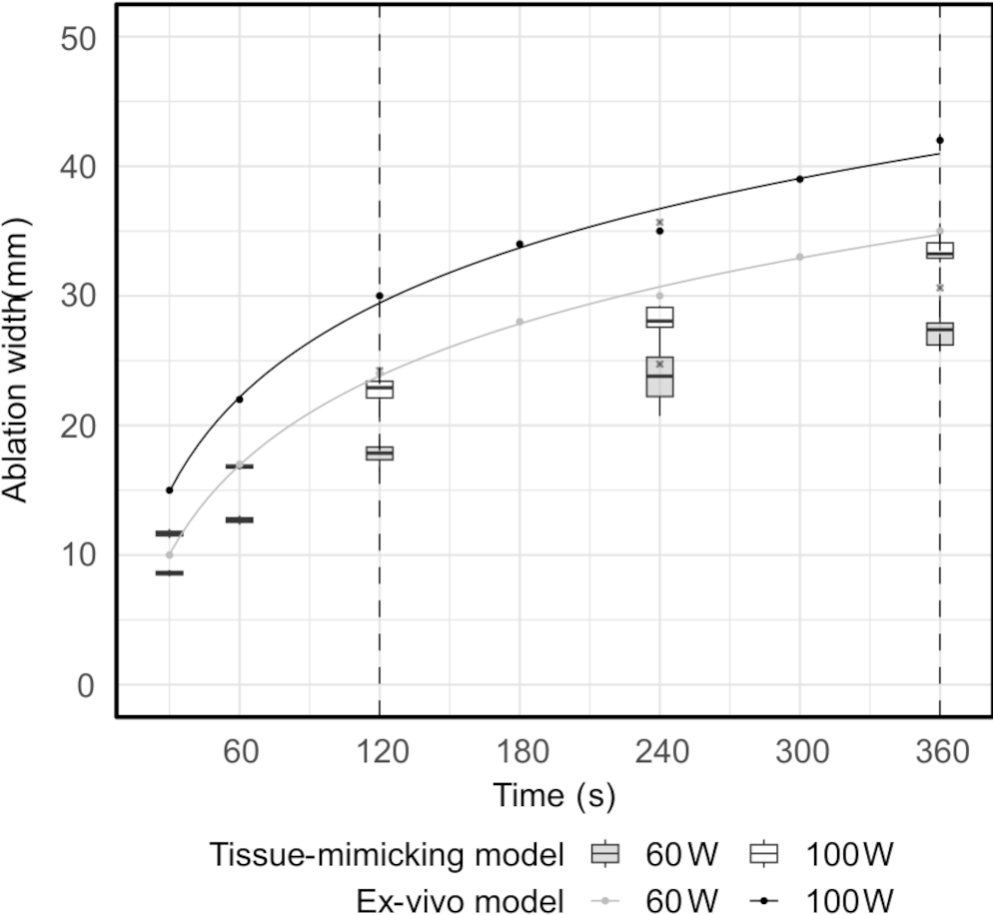
Ablation width measured in the tissue-mimicking model versus in the *ex-vivo* model, as provided in the ablation device manufacturer’s catalogue (range between the dashed lines). The solid lines represent fitted logarithmic regression models.

Standard ablation shapes deviated from an *ideal* ellipsoid. The shapes displayed extrusions along the lateral edges in four cases and noticeable indents in the central proximal areas in all cases (**Figures 7A, B**). Similarly, extrusions along the contour edges were also noted in six configurable ablation shapes (**Figures 7C, D**). Furthermore, standard ablation profiles produced smaller ablation volumes in the tissue-mimicking model compared to ablation volumes reported by the ablation device manufacturer in ex-vivo animal tissue (**Figure 8**). On average, the relative differences were 21% for the ablation width, 25% for the ablation length, and 52% for the estimated ablation volume, where the ablation volume was estimated from the revolved cross-sections. Lastly, the relative difference of the sphericity indices was 1–11% across different tissue-mimicking ablation volumes compared to the ex-vivo measurement catalogue.

## Discussion

This study investigates the repeatability of the novel robotic approach for configurable ablation tailored to distinct tumor shapes in the liver (19). The approach was introduced as a potential solution to treatment of irregularly shaped tumors, including those in challenging intrahepatic locations, with minimal thermal damage outside the ablative margin. The uncustomary technique of *dynamic* and *moving* thermal ablation employed for shape configuration has not yet been introduced in clinical treatment of liver tumors.

The repeatability of configurable ablation shapes was observed to be noninferior to the standard ablation shapes in the tissue-mimicking liver model by less than the predefined noninferiority margin of 1 mm. Furthermore, the 95% confidence interval for the differences in median repeatability between the two groups was significantly below zero, indicating that the proposed technique is as consistent or more consistent than the standard technique. Regarding safety, the maximal shape repeatability of 3 mm for both groups was below the reported safety distance required to prevent thermal injury (5–10 mm) (24).

Regarding irregularities in created ablation shapes (**Figure 7**), while lateral extrusions could be attributed to inhomogeneous gel formation of the tissue-mimicking model, proximal indents were likely caused by ablation probe ineffectiveness. Similarly to the heat-sink effect observed near vascular structures, the cooling liquid running through the ablation probe was presumably the cause of energy dissipation, creating indents on the top of the ablation contours. This effect was more pronounced in the standard than in the configurable shapes. When the ablation probe was static for a longer period, the thermal zone was localized around the probe. The influence of heating, however, weakened further away from the energy source. Consequently, it is presumed that the cooling of the probe had a pronounced effect on the ablation shape in the area around the probe and further away from the source. On the other hand, when the probe was moving, the effect of cooling did not play a role on the ablation shape formation, since the energy source was in motion. Evidently, the effect of applied ablation profile on ablation shape configuration was multifold. Slow and controlled retraction speeds, continuous and stable energy delivery and adequate probe cooling were essential to create the desired shapes. Moreover, it was possible to configure the shapes without continuous movement of the probe, i.e. in a stepwise motion, likely due to heat conduction which was propagating upwards in the direction of the probe and therefore connecting the “steps”.

The tissue-mimicking model used in this study represented a somewhat *ideal* medium due to its homogenous property and simplicity of analysis. The standard tissue model, applied in most cases of commercial ablation devices was an ex-vivo animal liver (porcine, bovine), as it is more representable of the living human tissue. Nonetheless, it was presumed that the complexity of the model could only affect the scale of repeatability of both techniques (*standard* vs. *configurable*), rather than their relative difference. Differences in material characteristics, such as thermal conductivity, diffusivity, and coagulation properties, could be the cause of discrepancy in ablation dimensions and sphericity between the models (**Figure 8**). Unknown history of the ex-vivo samples, including factors such as the temperature, freshness, and size, may also play a role in explaining the observed variations.

In comparison to the proposed configuration model, in clinical practice, there are two existing techniques for ablation shape configuration, overlapping ablation technique (OAT) and moving-shot technique (MST), which have been successfully deployed. The OAT primarily serves to ablate larger tumors, if they are not possible to eliminate with a single ablation volume. Custom ablation shapes can be created in the process; however, they require thorough planning and accurate probe positioning, as many ablation volumes are needed to be tailored to a tumor shape (25). However, this technique is not suitable for intricate patient cases as it does not allow precise ablation control. On the other hand, the MST has been introduced with the specific reason of uncompromised safety from collateral thermal injury. Nonetheless, this technique has only been effective for treatment of thyroid nodules since the thyroid gland is easier to access with an ablation probe than the liver and can be ablated using ultrasound guidance (18). This quality particularly enables the physicians to monitor thermal damage and prevent possible injury. Furthermore, due to convenient position close to the surface of the skin and small size of the tumor nodules, the ablation probe can be retracted manually with adequate dexterity. If MST were to be applied to a larger organ such as the liver, effective ablation control could not be achieved. The reason is that in the liver, thermal energy needed to be deposited in a single ablation *shot* would be much higher than in the thyroid, since the targeted volumes would be larger. Hence, manual execution would not be feasible because in order to deliver that higher amount of energy, the probe would need to move at very slow retraction speeds (< 1 mm/s).

Lastly, the absence of relevant clinical literature on the topic of treatment strategy in terms of different tumor shapes limits at times the decision-making process to technical specifications rather than patient-specific solutions. Current technical solutions of commercial thermal ablation devices narrow the applicability of thermal ablation treatment for liver tumors. Namely, the maximal ablation volume across all available microwave ablation devices measures 56 mm in width and 78 mm in length. Therefore, complete tumor coverage can be effectively achieved only on small spherical tumors with a sufficient 5–10 mm ablative margin.

Potential limitations of the current study include the unbalanced sample size and the assumption of independent measurements in the configurable shapes group. While the unbalanced sample size was considered in the sample size calculation, the use of a robust variability estimator aimed to mitigate the impact of underlying correlations among the measurements. Furthermore, it is estimated that the measurement uncertainty of ablation width and length in the tissue-mimicking model was between 1–2 mm, as there was no singular cut-off color intensity between ablated (magenta color) vs. non-ablated areas (off-white color). Unless the ablated area was differentiated by the mapped temperature values (21), it was not possible to set a clear-cut segmentation boundary corresponding to the area that reached the necrotic temperatures above 60°C. Further, the tissue-mimicking model lacked realistic depiction of the human physiology, thus more suitable models such as *in-vivo* animal or perfused ex-vivo animal models would be required as the following step of the preclinical evaluation phase to test the safety and the efficacy of the proposed solution. Nonetheless, the model provided a valuable preclinical canvas to test the feasibility and the repeatability in a reproducible and rigid experimental manner. Lastly, potential clinical limitations in terms of safety and accuracy related to the breathing motion would be compensated with high-frequency jet ventilation (HFJV), a state-of-the-art technique advisably used with the aforementioned decision support system (26–28).

## Conclusion

This study concludes that the repeatability of configurable ablation shapes was observed to be noninferior to the standard ablation shapes, within the predefined noninferiority margin of 1 mm. Moreover, the effectiveness of the proposed robotic platform and configuration model for thermal ablation underscores promising advancements towards enhanced treatment standardization and personalization tailored to individual clinical scenarios. This may broaden the applicability of thermal ablation to distinct tumor cases, which are deemed challenging by the standard ablation techniques. In this perspective, a fully integrated platform, with direct feedback from the ablation system to the robotic navigation system, could provide automated control of ablation power, time, and probe speed. In addition to the existing solutions for treatment planning, automatic segmentation, and direct quantitative assessment of ablation margins, the proposed treatment model could provide a complete solution for a standardized thermal ablation treatment even in complex tumor cases. As a next step, *in-vivo* validation is needed for evaluation of the clinical implications of the proposed treatment model.

## Data Availability

The raw data supporting the conclusions of this article will be made available by the authors, without undue reservation by the authors upon request.
